# Multi-Sensorimotor Training Improves Proprioception and Balance in Subacute Stroke Patients: A Randomized Controlled Pilot Trial

**DOI:** 10.3389/fneur.2019.00157

**Published:** 2019-03-01

**Authors:** Chaegil Lim

**Affiliations:** Department of Physical Therapy, College of Health Science, Gachon University, Incheon, South Korea

**Keywords:** sensorimotor training, proprioception, balance, stroke, hemiplegia

## Abstract

**Introduction:** The objective was to determine whether advanced rehabilitation therapy combined with conventional rehabilitation therapy consisting of sensorimotor exercises would be superior to usual treadmill training for proprioception variation and balance ability in subacute stroke patients.

**Methods:** Thirty subjects (post-stroke time period: 3.96 ± 1.19 months) were randomly assigned to either a multi-sensorimotor training group (*n* = 19) or a treadmill training group (*n* = 18). Both groups first performed conventional physical therapy for 30 min, after which the multi-sensorimotor training group performed multi-sensorimotor training for 30 min, and the treadmill training group performed treadmill gait training for 30 min. Both groups performed the therapeutic interventions 5 days per week for 8 weeks. The primary outcome (proprioception variation) was evaluated using an acryl panel and electrogoniometer. The secondary outcome (balance ability) was measured using the Biodex Balance system before intervention and after 8 weeks.

**Results:** The multi-sensorimotor training and treadmill training groups showed significant improvement in proprioception variation and balance (overall, A-P and M-L) (all *P* < 0.05). In particular, the multi-sensorimotor training group showed more significant differences in proprioception variation (*P* = 0.002) and anterior-posterior (A-P) balance ability (*P* = 0.033) than the treadmill training group.

**Conclusions:** The multi-sensorimotor training program performed on multiple types of sensory input had a beneficial effect on proprioception sense in the paretic lower limb and A-P balance. A large-scale randomized controlled study is needed to prove the effect of this training.

**Clinical Trial Registration:** https://cris.nih.go.kr/cris/, identifier KCT0003097.

## Introduction

Impaired sensory and functional balance abilities after strokes often make it difficult for patients to return to their activities of daily living (ADL), thus creating a potential burden to family members and society. Approximately 50% of stroke patients experience sensory impairment. Occasionally, these neurological disorders are accompanied by aphasia, hemianopsia, or neglect. For over 50% of patients, sensory defects present on the affected side (1).

Sixty-five percent of stroke patients experience impaired tactile and protective responses, including proprioceptive sensations (1). As a result, stroke patients are less able to transmit information to the brain and spinal cord regarding muscle strength, pressure, joint position, and muscle length, which are required to maintain posture and can be detected in various joints on the paralyzed side (2).

Postural control involves biomechanical constraints, cognitive processing, control of dynamic, orientation in space, movement strategies, and sensory strategies. Sensory information from somatosensory, vestibular, and visual systems is then integrated, and the relative weights placed on each of these inputs are dependent on the goals of the movement task and the environmental context (3). Stroke patients typically have decreased balance reaction times, postural sway strategies, and impaired body weight support of the hemiparetic limb (4). Balance impairment ranks first among stroke disorders. Decreased muscle power, coordination, and sensory make it difficult to maintain balance (5). Furthermore, decreased balance not only potentially increases the risk of falls and femoral neck fracture but also decreases the ability to perform physical activity (6).

In general, stroke patients regain physical ability from sensory stimulation and mass motor exercise or task-oriented practice facilitating neural plasticity (7). Rehabilitation therapy programs can be classified as either conventional or advanced programs according to the theoretical background and clinical trials (8). Conventional rehabilitation therapy programs include the Bobath concept, Brunnstrom approach, proprioceptive neuromuscular facilitation (PNF), and functional strengthening approaches that emphasize motor learning and control, functional activity, or muscle strengthening (9). Regular and repetitive therapies involve sensory input with visual, verbal, tactile, cutaneous, proprioceptive, and auditory assistance for clinical stroke rehabilitation (8). Advanced rehabilitation therapy programs include electrical stimulation (10), robotic therapy (11), and virtual reality (12) for proprioceptive, tactile, visual, and auditory assistance in specific interventions based on neuroscientific evidence (9). Previous studies have recommended trunk control by training on a vibration board (13) and reactive balance training through perturbation in spastic diplegia cerebral palsy (14). According to Aman et al. (15), using a muscle spindle to stimulate active and passive sensorimotor training affects postural control and balance.

Depending on the stroke patient's ability and recovery stage, appropriate advanced rehabilitation therapy combined with conventional rehabilitation therapy consisting of sensorimotor exercises can provide multiple types of sensory input to assist in recovery after stroke (8). As Smania et al. described (16), with a specific training program based on weight transfer and balance exercise performed under different conditions of manipulation of sensory inputs, chronic stroke patients achieved significant improvement in their ability to maintain balance control. Sensorimotor training progressively improves the ability to re-weight and integrate sensory inputs to control balance, even in conditions where somatosensory input has been altered, and to avoid falls (17). However, to our knowledge, previous studies that have targeted multi-sensory input training for stroke patients are very limited.

It was hypothesized that advanced rehabilitation therapy combined with conventional rehabilitation therapy consisting of sensorimotor exercises would be superior to the usual treadmill training for proprioception variation and balance ability in subacute stroke patients.

## Methodology

### Setting, Study Design, and Participants

This study was a two-arm, parallel, and randomized controlled pilot trial with concealed allocation and participants as well as blinding for the researcher and assistants. All procedures from this study were approved by the Institutional Review Board of Gachon University (IRB No.: 1044396-201803-HR-068-01) and registered at Clinical Research Information Service (CRiS), Republic of Korea (KCT0003097) and all participants signed informed consent prior to beginning the study. In addition, this study conforms to all CONSORT guidelines.

### Procedures

Stroke patients were recruited from a rehabilitation hospital in Incheon, South Korea. Participants (inpatient or outpatient) 50–71 years of age who experienced their first stroke were enrolled in this study if it had been 6 months or less since the unilateral hemisphere stroke, they could walk for 30 s or more (regardless of using assistance), and they completed the mini-mental state examination (MMSE) with a score of 24 or more. All patients had experienced stroke as defined by computed tomography (CT) or magnetic resonance imaging (MRI). Exclusion criteria consisted of the presence of a cognitive disorder, visual disorder and severe unilateral neglect, cardiorespiratory disorder (with cardiac pacemaker), concurrent neurological disease (e.g., Parkinson disease), orthopedic intervention, having over G2 on the Modified Ashworth Scale and receiving botulinum toxin injections for spasticity within the past 6 months.

### Randomization and Masking

Baseline measurements of patients' abilities were performed prior to randomization. Subsequently, each participant was allocated to one of the two groups via allocation codes included in consecutively numbered, sealed, opaque envelopes. Simple randomization was conducted using Microsoft Excel for Windows software (Microsoft Corporation, Redmond, WA, USA) by a researcher who was not involved in participant recruitment. To ensure masking, protocols and intervention order were not revealed to clinical evaluators. Intervention allocation was recorded in a password-protected document to maintain blinding. All data were measured by the same blinded physical therapist before the intervention and at the end of the 8 weeks intervention period.

### Interventions Procedures

All groups performed conventional rehabilitation therapy for 30 min. Then, the multi-sensorimotor training group performed Stabilize-T and Reha-Bar (Pedalo®; Holz-Hoerz GmbH, Münsingen, Germany) exercises (18) with transcutaneous electrical nerve stimulation (TENS) for 30 min. The treadmill training group participated in treadmill gait training with placebo TENS (with only adhesive TENS electrodes) for 30 min (19). We applied one of these methods to the patients in accordance with their ability. In all interventions and assessments, if patients complained of discomfort, they were told to stop immediately and take a rest. Both groups received an intervention for 5 days per week for 8 consecutive weeks.

#### Conventional Rehabilitation Therapy

Conventional rehabilitation therapy, such as the Bobath approach or the PNF approach, was performed for 30 min. The patients were in the supine position, the trunk and upper and lower parts of the back were aligned, stability of the trunk was ensured, and limb movements of the hip joint, knee joint, and ankle joint were induced. Subsequently, the subjects maintained stability in the trunk and repeated flexion and extension of the lower limbs. A skilled physical therapist provided assistance so that alignment was not altered. The backward tilt of the pelvis involved the use of the pelvis and back muscles. The sitting exercises were performed for smooth pelvic movement and co-contraction of the hamstrings and quadriceps muscles by adjusting the height using a Bobath table to control muscle strength. We performed weight support training on the left and the right in the standing position and one-leg position.

#### Multi-Sensorimotor Training Program

**Figure 1:**
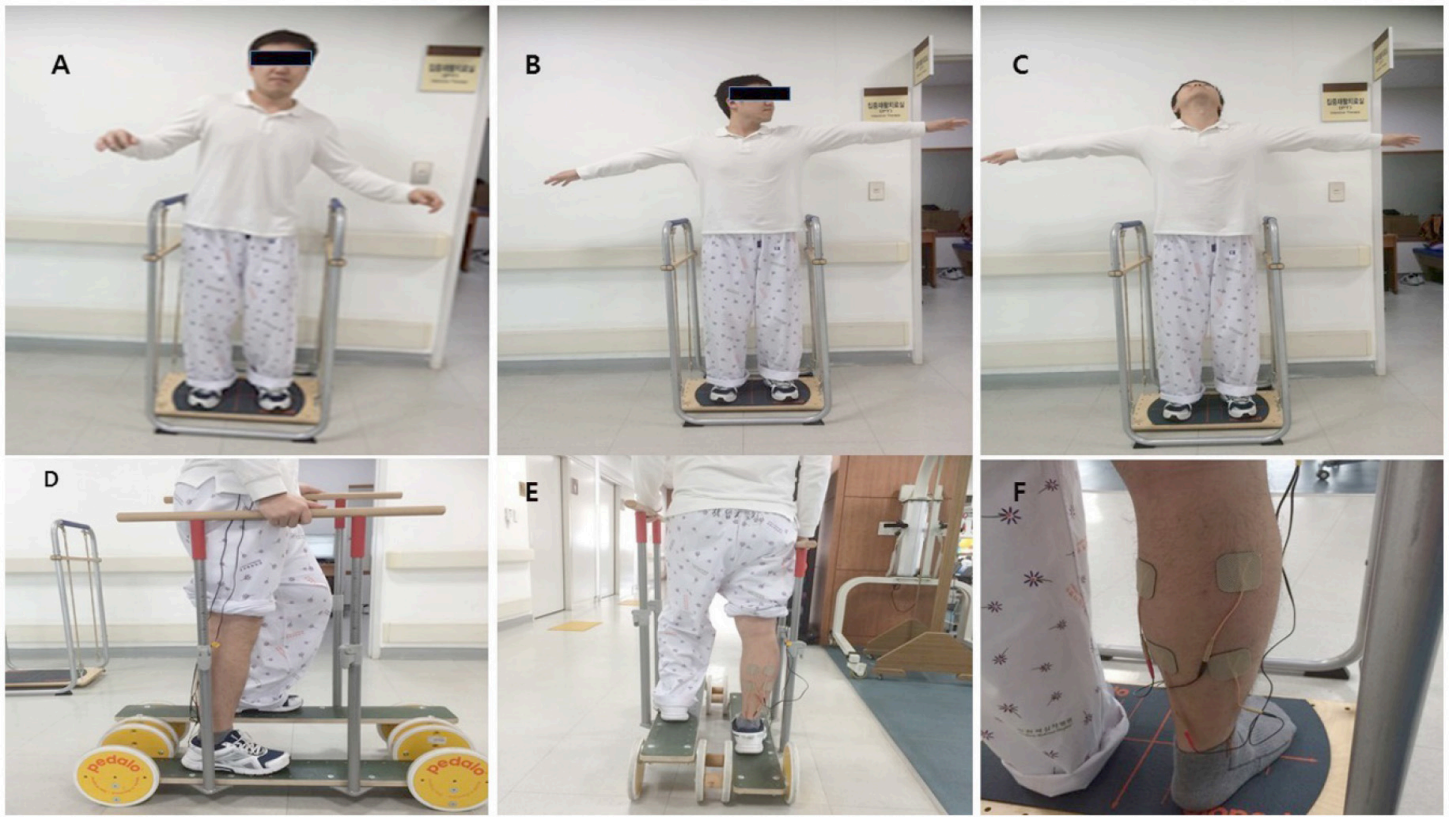
The Stabilize T and Reha-Bar with transcutaneous electrical nerve stimulation (**A–C**: Stabilize T exercise, **D,E**: Reha-Bar exercise, **F**: TENS).

The multi-sensorimotor training program included Stabilize-T and Reha-Bar (Pedalo®) (Figure 1) exercises (18) with TENS (19). The electrical stimulation therapy was delivered by a dual-channel TENS unit during the bipolar balanced phase, with a pulse duration of 200 μs and frequency of 100 Hz (TENS 7000TM; Koalaty Products Inc, Florida, USA). Adhesive TENS electrodes (5 × 5 cm) were fastened to the paralyzed medial and lateral motor points of the gastrocnemius muscles. Under stimulation conditions, the TENS intensity was adjusted before the start of measurements in increments of 0.01 mA and set at the subsensory threshold of each patient (19). Stabilize-T exercises improved sensory input (proprioceptive and tactile) to help control the posture balance of the locomotor activity (18).

The exercises were performed as follows. First, patients looked ahead while standing with the knee slightly bent on the Stabilize-T (start posture). They closed their eyes for 15 s, opened their eyes for 10 s, and maintained balance for 30 s. After patients stood on the Reha-Bar, they pedaled up and down and rotated the wheels to move forward while holding the safety bar with both hands with the therapist's assistance. Second, patients posed in the same position as the first posture. Then, patients slowly abducted the arms and rotated the neck to the left, center, and right for 30 s each and maintained balance for 30 s. Then, after patients stood on the Reha-Bar again, they pedaled up and down and rotated the wheels to move forward while holding the safety bar with both hands without the therapist's assistance. Third, patients posed in the same position as the first posture. Patients abducted the arms and looked upward for 10 s. Patients repeated the neck extension exercise three times and maintained balance for 30 s. After patients stood on the Reha-bar, they pedaled up and down and rotated the wheels to move forward and backward while holding the safety bar with both hands without the therapist's assistance. The first exercise was the easiest and the third exercise was the most difficult. These exercises indicated the abilities of the patients in this study (18).

#### Treadmill Gait Training Program

The treadmill gait training program involved gait with placebo TENS for 30 min at the paralyzed medial and lateral motor points of the gastrocnemius muscle. Patients participated 5 days per week for 8 weeks (20). Gait training started at low intensity for 10 min (50% heart rate reserve [HRR]). The exercise duration increased 5 min every 2 weeks, and the exercise intensity increased 5% HRR every 2 weeks. The final goal was 30 min at 70% HRR (21).

### Outcome Measurements

Baseline general characteristics were collected through file audit and self-reporting. The primary outcomes were the changes in the proprioception sense. The secondary outcomes were the changes in the balance ability. Primary and secondary outcome measures were collected in the hospital after randomization and after 8 weeks in the same place. Researchers masked to treatment allocation collected and entered all data. The physical therapist recorded their intervention recommendations. A research assistant conducted a blinded content analysis of the recommendations to provide relevant descriptive categories for analysis.

**Figure 2:**
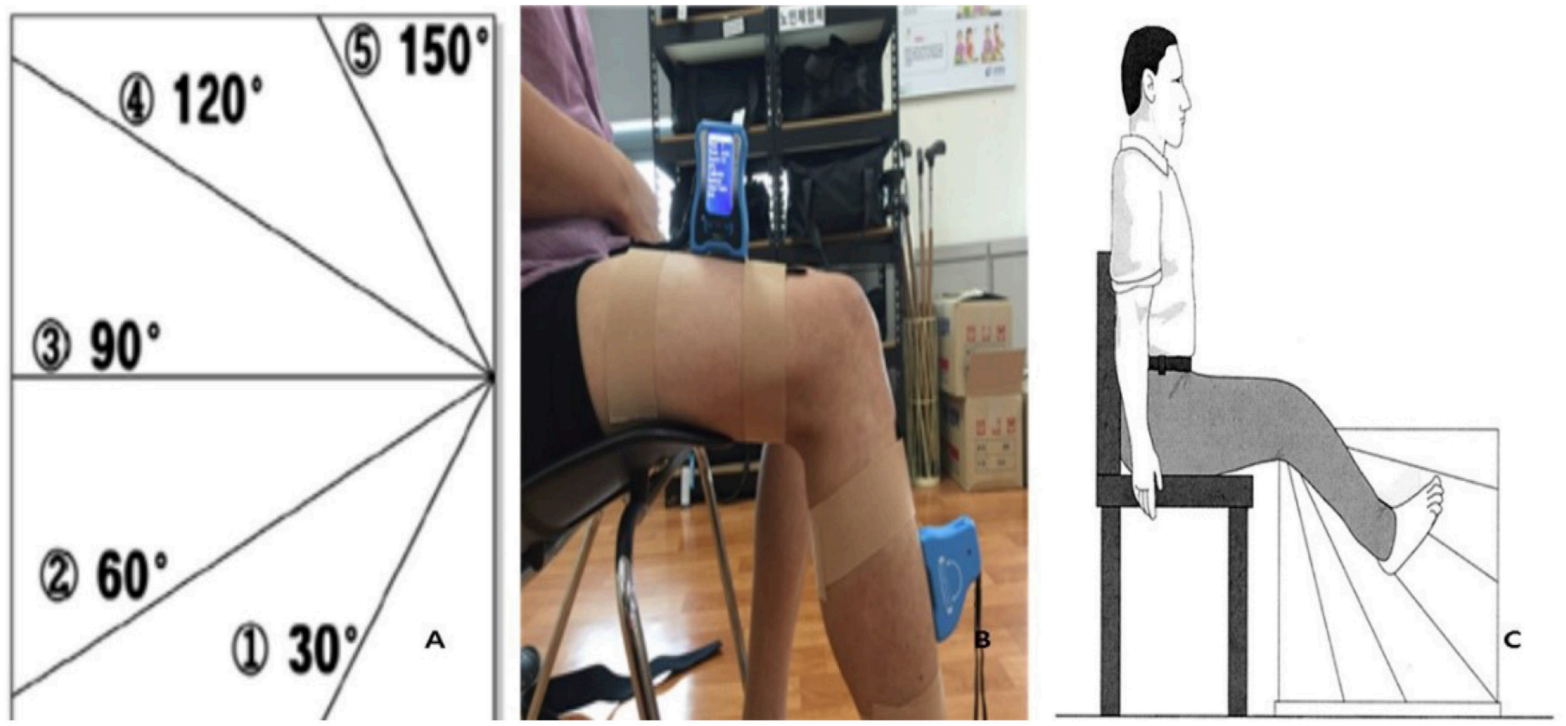
Proprioception test (**A**: Acryl panel, **B**: Electrogoniometer, **C**: Test position). Reproduced with permission from Lord et al. (22).

Proprioception variation was assessed using an acryl panel (60 × 60 × 1 cm) and electrogoniometer (JTECH Medical DUALER IQ PRO; Salt Lake City, UT, USA). In the sitting position, patients were asked to close their eyes and aligned their lower limbs on both sides of a clear acrylic panel. Then, the electrogoniometer was attached to the quadriceps and anterior tibia (Figure 2) (23). Each trial was performed for 10 s, and resting was required between trials to avoid fatigue and learning effects. The angle of the affected limb was measured after patients memorized the position of the unaffected limb indicated by the therapist. An average of five tests were recorded after two practice sessions (22).

Balance abilities were measured by the Biodex Balance system (BBS; Biodex Medical System, Inc., Shirley, NY, USA). This device focuses on the proprioceptive neuromuscular functions that appear to affect dynamic joint and postural stability. During postural balance testing, the patient's ability to control the platform's tilt angle was evaluated as a deviation from the center. The BBS software (Biodex version 1.08, Biodex, Inc.) presented the degree of deviation in each axis and provided an average sway score. The score levels ranged from 1 (low stability) to 8 (high stability) (24). During three trials, each test was performed for 20 s, followed by a 10 min resting period (25).

### Sample Size Estimation

We estimated that the minimal acceptable sample size would be 21 patients per group to achieve a power of 0.8 with a significance level (α) of 0.05 using a one-sided two sample *t*-test by G^*^Power 3.1.9.1 software for Windows (Uiversität Kiel, Germany). It was decided that 29 patients would be necessary based on an inter-groups difference in proprioception improvement in a previous trial (26).

### Statistical Analysis

SPSS 23.0 software for Windows 7 (IBM Corp., Armonk, NY, USA) was used to analyze the data. Data were summarized using means and standard deviation (SD). The normality of the parameter distributions were assessed using the Shapiro-Wilk test. If the data show a normal distribution, data were expressed as the mean ± standard deviation (continuous data) or percentage (categorical data), and parametric tests such as independent samples *t*-test or the χ^2^ test were used to compare the general characteristics of the two groups. For a within-group comparison, a paired *t*-test was used and comparison between the two independent groups (multi-sensorimotor group and treadmill group) was accomplished with an independent *t*-test. The level of significance was set at α = 0.05.

## Results

**Figure 3:**
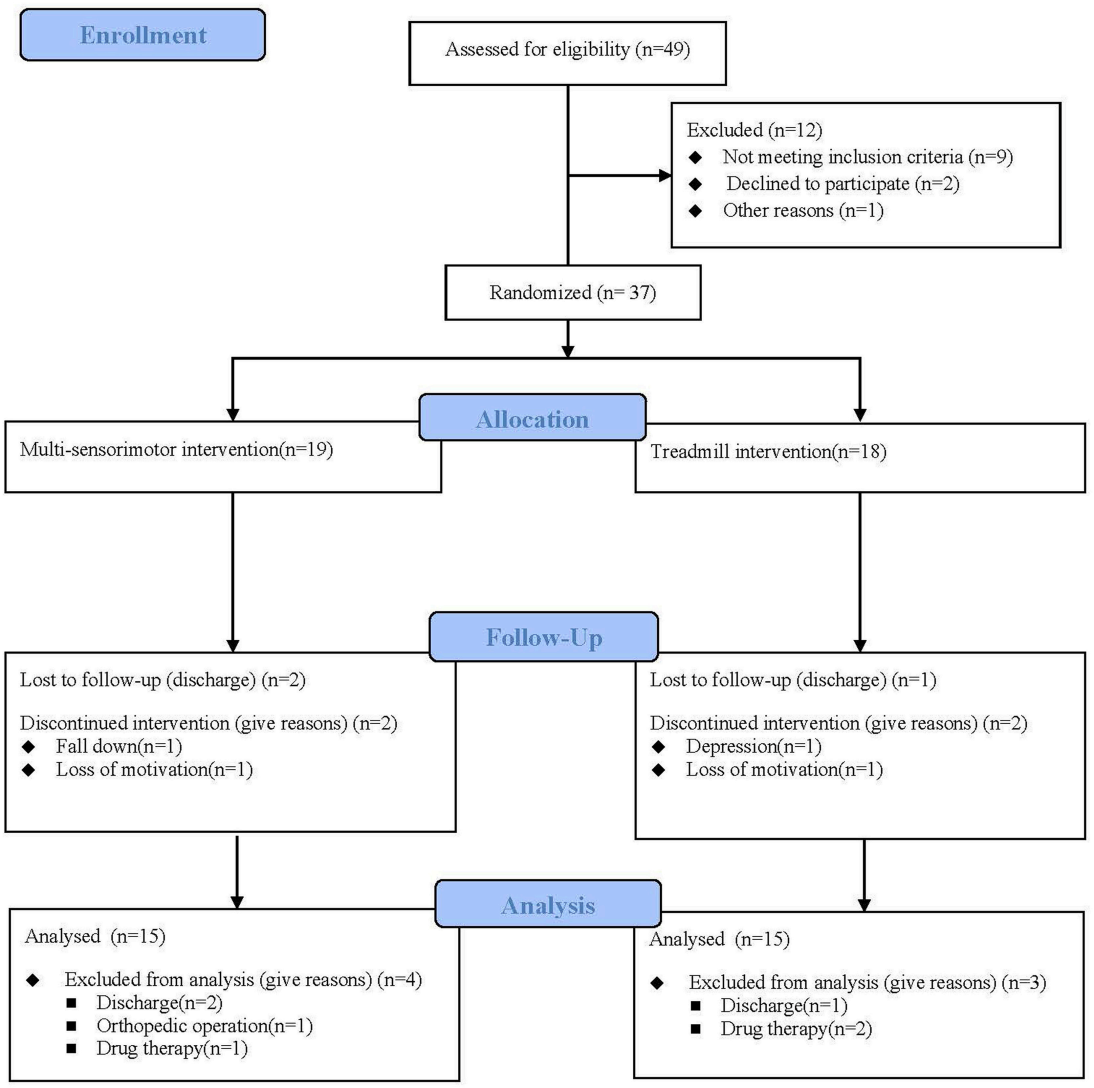
Flow diagram of this study. Thirty-seven individuals were enrolled in the study and were randomly assigned to the multi-sensorimotor group (*n* = 19) or the treadmill group (*n* = 18).

Between August 2017 and April 2018, a total of 49 stroke patients were admitted to the hospital and 37 fulfilled the inclusion criteria. Participants were randomly assigned to the multi-sensorimotor training group (*n* = 19) or the treadmill gait training group (*n* = 18). Of 37 participants who began the study, 30 (81%) completed it (Figure 3). A total of 7 patients (19%) were lost to follow-up or discontinued intervention. The general baseline characteristics of the participants of the two groups are described in Table 1. Recorded characteristics included gender, age, height, weight, lesion side, lesion type, and post-stroke duration. The mean ± SD age of the participants was 60.80 ± 7.03 years, and the mean ± SD post-stroke duration was 3.96 ± 1.19 months. Baseline demographic characteristics such as gender (males/females, 11/8 vs. 10/8), age (62.00 ± 7.30 vs. 59.61 ± 6.77 years), lesion type (ischemic/hemorrhagic, 13/6 vs. 11/7), lesion side (right/left;7/12 vs. 7/11), and post stroke-duration (4.05 ± 1.12 vs. 3.88 ± 1.27 months) were not significantly different between the multi-sensorimotor training group and the treadmill training group (*P* > 0.05).

**Table 1:** General characteristics of the two groups by randomization assignment.

	**Multi-sensorimotor group(*n* = 19)**	**Treadmill group (*n* = 18)**	***P***
Gender (male/female)	11:8	10:8	0.88^a^
Age (years)	62.00 ± 7.30	59.61 ± 6.77	0.30^b^
Height (cm)	168.26 ± 4.95	165.38 ± 5.19	0.94^b^
Weight (kg)	63.05 ± 8.66	64.88 ± 7.52	0.49^b^
Lesion side (right/left)	7:12	7:11	0.82^a^
Lesion type (ischemic/hemorrhage)	13:6	11:7	0.64^a^
Post-stroke duration (month)	4.05 ± 1.12	3.88 ± 1.27	0.68^b^
**Balance (Score)**			
Overall	2.90 ± 0.95	2.96 ± 1.06	0.85^b^
A-P	2.10 ± 0.94	2.11 ± 0.88	0.97^b^
M-L	1.97 ± 1.03	1.71 ± 0.71	0.38^b^
Proprioception (degree)	11.89 ± 3.64	11.16 ± 3.65	0.54^b^

*Data are expressed as mean ± SD or n (%)*.

a The P-value was obtained using a χ^2.^

b *The P-value was obtained using an independent t-tests*.

The multi-sensorimotor training groups had improved proprioception after rehabilitative training compared to the treadmill training group (*P* < 0.001; effect size = 0.55; power 71%). Additionally, the A-P balance ability score of the multi-sensorimotor training group improved more than that of the treadmill gait training group (*P* = 0.03; effect size = 0.39; power 65%). Both groups had significantly improved balance ability scores (overall, anterior-posterior [A-P], and medial-lateral [M-P]) (*P* < 0.05) after intervention (Table 2).

**Table 2:** Changes in balance and proprioception within each group and between the two groups.

			**Intragroup**		
**Variance**			**Multi-sensorimotor group(*n* = 15)**	**Treadmill group (*n* = 15)**	**Intergroup (*p*)**
Balance (score)	Overall	Pre	2.86 ± 0.85	3.0 ± 1.13	
		Post	1.71 ± 0.61	2.14 ± 1.11	
		*P*	0.000^a^	0.000^a^	
		Post-Pre	−1.15 ± 0.55	−0.86 ± 0.72	0.223
	A-P	Pre	2.13 ± 0.90	2.07 ± 0.84	
		Post	1.10 ± 0.79	1.55 ± 0.77	
		*P*	0.000^a^	0.004^a^	
		Post-Pre	−1.02 ± 0.64	−0.52 ± 0.58	0.033^b^
	M-L	Pre	1.92 ± 0.92	1.66 ± 0.76	
		Post	1.27 ± 0.62	1.16 ± 0.72	
		*P*	0.000^a^	0.017^a^	
		Post-Pre	−0.64 ± 0.45	−0.50 ± 0.71	0.507
Proprioception (degree)		Pre	12.20 ± 3.60	11.06 ± 3.97	
		Post	7.00 ± 2.53	8.53 ± 2.94	
		*P*	0.000^a^	0.000^a^	
		Post-Pre	−5.20 ± 2.45	−2.53 ± 1.72	0.002^b^

*Data are presented as mean ± SD*.

a *P < 0.05. The P-value was obtained using a paired t-test*.

b *P < 0.05. The P-value was obtained using an independent t-test*.

## Discussion

This study found that proprioception and A-P balance ability significantly improved in those in the multi-sensorimotor training program compared to those in the treadmill gait training group. This method using vibration, tactile, proprioception, and vestibular senses was effective for improving balance, especially in the multi-sensorimotor training group.

In the previous study, Moreside et al. (27) measured the activities of multiple trunk muscles by using electromyography, while the subjects performed horizontal-vibration exercises and showed that the activities of the internal oblique abdominal muscle and external oblique abdominal muscle were the highest. In contrast to this study, the multi-sensorimotor training involved Stabilize-T exercises from the all-direction vibration because the improvements were activated in the internal and external oblique abdominal muscles, the erector spinae muscle, latissimus dorsi muscle, and rectus abdominis muscle. The activation of these trunk muscles suggested that all-direction vibration stimulates improved balance ability (28). Additionally, previous studies reported that using vibration with the eyes closed improved the balance ability of healthy elderly participants because their balance ability had decreased more than that of healthy non-elderly subjects (4).

Neurophysiological observations suggest that changes in the process of sensorimotor integration do not occur at the peripheral level but depend on abnormal central processing of sensory input (29). These study results showed that proprioceptive sensory changes improved by 43% and 23% through multi-sensorimotor training and treadmill gait training, respectively. The multi-sensorimotor program, which comprised the neurological summation of the proprioceptive stimulus, was more effective than the other program. Proprioception involved electrical stimulation of the paralyzed calf muscles for 30 min during the intervention period; tactile, vestibular, and kinesthetic sensations were stimulated through the Stabilizer-T and Reha-Bar exercises. In addition, the muscle response needed to control the postural sway improved. Therefore, it was more effective for improving the A-P balance ability. In the multi-sensorimotor training group, the A-P balance ability was improved more effectively than the M-L balance ability. It can be inferred that the vestibular coordination exercises using the pedal tool effectively enhanced the peri-articular sensations of the surrounding soft tissues and muscles to control A-P balance.

The most direct cause of the balance recovery through TENS is an increase in somatosensory information from the lower limbs because the sensory stimulation through TENS increases the flow of somatic sensation rising from the lower limb that maintains and controls the standing posture (30). Additionally, Golaszewski et al. (31) reported that electrical stimulation increased signaling in the pre- and post-central gyri after cutaneous stimulation. The inferior parietal lobule was also activated in both hemispheres, and it is feasible that additional afferent stimulation might trigger the remaining plastic capacity for sensorimotor reorganization in the brain and might thus facilitate functional recovery in chronic stroke.

We also noted that there are significant improvements in overall and M-L balance after intervention in the multi-sensorimotor training group, but there were no intergroup differences. This may be because the improvements were observed in treadmill training groups by conventional therapy and treadmill training in the subacute phase.

This study has several limitations. First, 30 participants completed the study, which was insufficient to identify inter-group changes. Second, the long-term effects of the training could not be confirmed. In addition, we could not exclude the learning effect for each evaluation system. Third, we could not evaluate the postural control in ADL and the fear of falling. Finally, the level of the ankle muscle activity could not be directly proven. Therefore, future studies need to include more participants. In addition, a method that can directly quantify ankle and trunk muscle strength, such as electromyography (EMG) activity, should also be attempted, and the studies should be designed to explore whether the training effects are still present months after the experiment.

## Conclusion

This study provided evidence that combined rehabilitation methods significantly enhanced the proprioception and balance ability during the subacute phase of recovery after stroke. Therapists have an important role in the achievement of maximum benefits throughout the rehabilitation process after stroke. The optimal intensity and duration of specific interventions have been systematically evaluated, and it has been indicated that combining valuable training exercises for multiple senses is believed to be a good method for facilitating the restoration of proprioception and balance ability.

## Author Contributions

CL made substantial contributions to conception and design, acquisition of data (two research assistants helped), data analysis, interpreting the data, drafting the article, and revising it critically for important intellectual content and final approval of the version to be submitted.

### Conflict of Interest Statement

The author declares that the research was conducted in the absence of any commercial or financial relationships that could be construed as a potential conflict of interest.
